# Targeted medical examinations for workers exposed to fumigants

**DOI:** 10.1186/s12995-022-00361-3

**Published:** 2022-10-28

**Authors:** Zeenathnisa Mougammadou Aribou, Wee Tong Ng

**Affiliations:** Saw Swee Hock School of Public Health, National University of Singapore, 12 Science Drive 2, #10-01, 117549 Singapore, Singapore

**Keywords:** Chronic fumigant toxicity, Chronic fumigant exposure, Occupational Health Screening, Occupational Health Surveillance, Methyl Bromide, Hydrogen Phosphide, Hydrogen cyanide, Sulfuryl Fluoride

## Abstract

Fumigants are gaseous pesticides or biocides which eradicate pests by suffocation or poisoning. Worker exposure to fumigants is mainly via inhalation, followed by dermal contact and ingestion, leading to various acute and chronic health effects. Implementation of appropriate workplace controls such as adequate ventilation, training and personal protective equipment ensure that exposure to fumigants are kept to the lowest level as practically possible. In addition, routine medical examinations also allow for doctors to identify and manage possible exposure to fumigants and ascertain workers’ fitness to work.

While management guidelines after an acute exposure to such fumigants is clear and consistent, the guidelines on routine medical examination for fumigators is sparse. Components of the medical examinations vary according to the fumigant, workers are exposed to and its chronic health effects. Hence, this paper highlights the health hazards of commonly utilised fumigants; Methyl Bromide, Hydrogen Cyanide, Hydrogen Phosphide and Sulfuryl Fluoride; and outlines the guidance for routine medical examinations for exposed fumigators.

## Background

Fumigants are gaseous pesticides or biocides which eradicate pests by suffocation or poisoning [1]. They can be applied to stored products, antiques, furniture, vessels, and containers for treatment. Such targeted treatment is carried out in a controlled environment, in which an enclosed space such as fumigation chambers or temporarily created containment is filled with fumigants [1, 2]. Fumigants have various advantages over other control methods as they does not require any behaviour or action of the target organism for eradication [3]. They also penetrate cracks, crevices, and some packaging material. As there are different types of fumigants, the choice of fumigant will be determined by the target organism, location and treated commodity [3].

Worker exposure to fumigants is mainly via inhalation, followed by dermal contact and ingestion [1]. Fumigants are toxic chemicals, and exposure can result in harmful health effects. The toxicity of a health hazard can be divided into acute (short term) or chronic (long term) effects. Acute health effects have a short latency period and usually occur post-exposure to high concentrations of fumigants. Such health effects also depend on the exposure intensity and can be classified into mild, moderate, and severe exposure. Chronic health effects have a long latency period and can occur from repeated or continuous exposure over a period longer than three months [4]. Moreover, chronic health effects also constitute delayed clinical features that occur due to long latency can also arise from acute exposure [4].

In addition to the implementation of appropriate workplace controls such as adequate ventilation, routine medical examinations are essential in ensuring negative health implications from exposure to fumigants are kept to the lowest level as practically possible. Firstly, routine medical examinations mitigate health effects by regularly ascertaining fitness to work of fumigators. Fitness to work ensures that workers can carry out fumigation activity and do so in a safe manner without inducing harm to themselves or those around them. Secondly, routine medical examinations are pivotal in establishing evidence of chronic exposure, allowing for early diagnosis, management, and implementation of preventive measures. By doing so, routine medical examination are instrumental in ensuring workers are protected from the harmful effects of fumigants.

Components of the medical examinations vary according to the fumigant workers are exposed to and its chronic health effects. While management guidelines after an acute exposure is clear and consistent, the guidelines on routine medical examination for fumigators are sparse. This paper therefore aims to highlight the health hazards of Methyl Bromide, Hydrogen Cyanide, and Hydrogen Phosphide and outline guidance for periodic medical examinations for fumigators.

## Methods

Search terms such as “fumigants”, “methyl bromide”, “hydrogen phosphide” “hydrogen cyanide”, “sulfuryl fluoride”, “chronic health effects”, “chronic exposure” “examinations” and surveillance” were also searched in PubMed, Medline and the Cochrane Database of Systematic Reviews. This paper also referenced international guidelines from various countries and key occupational health and safety agencies such as Occupational Safety and Health Administration (OSHA), National Institute of Safety and Occupational Health (NIOSH), Health Safety Executive (HSE), Health Protection Agency (HPA), Enviromental Protection Agency (EPA) and the various Safety Data Sheet (SDS) of the fumigants on reported chronic effects.

Of note, while physicians should also screen for symptoms and signs of acute toxicity and prior episodes of acute exposures during routine medical examination, this paper will mainly focus on the chronic health hazards of Methyl Bromide, Hydrogen Cyanide, Hydrogen Phosphide and Sulfuryl Fluoride and outline guidance to identify chronic health hazards during routine medical examinations.

### Methyl bromide

Methyl Bromide (MeBr), also known as bromomethane or monobromomethane is a broad-spectrum pesticide with a long history of use as a fumigant. MeBr is a colourless gas at room temperature which has no odour or taste at low concentrations. At high concentrations, MeBr has a musty or fruity odour. In the gaseous state as fumigants, MeBr, is more than three times as dense as air and may collect in low spots and poorly ventilated places and can penetrate many substances such as concrete, leather and rubber [5–9].

MeBr is well absorbed via inhalation and is rapidly distributed to many tissues including lungs, adrenal glands, kidneys, liver, nasal turbinate, brain, testes, and adipose tissues. It can also penetrate the blood brain barrier, affecting the Central Nervous System (CNS) [5, 7, 9, 10]. It has a half-life of 12–14 days and is excreted in urine and via exhalation. Of note, a lachrymatory agent such as chloropicrin is often added to act to MeBr as a sensory warning agent. Chloropicrin has a pungent odour and causes eye irritation and its main health effects are lacrimation, irritation, cough and chest pain [5, 7, 11].

The Global Harmonised System of Classification and Labelling of Chemicals (GHS) classifies MeBr as toxic [12]. Fatalities have occurred amongst fumigators who were exposed to MeBr during application or who have prematurely re-entered fumigated premises [5]. MeBr methylates the sulfhydryl groups of enzymes and causes cellular disruption and reduced glutathione levels [7, 9–12]. Importantly, the cellular disruption occurs primarily in the CNS subsequently causing progressive neurological dysfunction. It has been reported that MeBr has a greater potential for toxicity than do other organic bromides because of its greater lipophilicity which provides increased access to the brain [18]. Of note, it is postulated that methanol, which is a metabolite of methyl bromide, may also contribute to the neurologic and visual effects in high levels of exposure [7, 9–12].

Chronic exposure to MeBr may result in neurological effects such as peripheral neuropathy, impaired gait, behavioural changes, mental confusion, lethargy, loss of coordination and muscle weakness [7, 9, 11, 13]. Visual impairment arising from optic atrophy has been reported following chronic exposure [9]. In fact, Chavez et al. reported that a fumigator who was chronically exposed to MeBr developed paraesthesia of the extremities, dysesthesias, and visual impairment secondary to optic atrophy [14]. MeBr is also listed as a skin irritant and so, repeated exposure may cause contact dermatitis [7, 9, 11]. However, there is no evidence that MeBr can induce.

sensitization [9, 15]. Of note, while chronic exposure to MeBr results in mild kidney or liver damage, elevated liver enzymes, proteinuria and haematuria is often seen after acute exposure only [4]. As such, liver function tests, renal panel and urine analysis have little value in evaluating for chronic exposure to MeBr.

A meta-analysis by Budnik et al. reported that overall exposure to MeBr is associated with an increased risk of prostate cancer with an odds ratio of 1.21 [16]. However, the International Agency for Research on Cancer has classified MeBr as a Group 3 carcinogen due to inadequate evidence to its carcinogenicity [6, 7, 9, 12]. No reproductive or teratogenic effects have been reported [6, 7, 9, 12].

Routine medical examinations should therefore identify workers who have medical conditions involving CNS, skin, liver, renal and respiratory system. For example, as MeBr is a skin irritant, workers with eczema can be at a higher risk of developing irritant reactions if exposed. The evaluation should also include a systemic evaluation of symptoms and signs to assess if workers have undiagnosed medical conditions such as kidney disease, chronic lung disease, skin conditions. Workers who have pre-existing medical conditions involving these systems should be counselled on the potential risk of exposure to MeBr and the increased susceptibility of developing health hazards.

Medical examinations should also entail (i) a skin examination looking for evidence of contact dermatitis, frostbite, or burns; and (ii) neurological examination particularly focusing on gait, peripheral and cerebellar systems looking for neurological features of chronic exposure. In patients with abnormal neurological features, physicians can consider performing laboratory investigations such as a full blood count (FBC) to rule out other causes of peripheral neuropathy. For example, megaloblastic anaemia secondary to B12 deficiency could result in peripheral neuropathy, thus negating Methyl Bromide as the cause of the deficit. In addition, direct fundoscopy and visual acuity should also be undertaken to look for optic atrophy. Such routine examinations aid in identifying chronic health effects that could have resulted from exposure at work. Likewise, workers who, upon examination are detected to have abnormal examination findings should be evaluated further to ascertain fitness to work (*refer to* Table 1). Of note, While blood bromide can be measured at the end of the shift at the end of the work week, it is only useful if it is done within 1 to 2 days following exposure and hence will not be a good indicator of chronic exposure. There is currently no recognised biological occupational exposure limit for urine bromide [11, 17].

**Table 1:** Overview of routine medical examinations

Fumigant	Routine medical examination
Methyl Bromide	● Medical history evaluating for past medical conditions or symptoms involving the following systems. ○ Neurological ○ Respiratory ○ Skin ○ Liver ○ Renal ● Examination ○ Skin - looking *for evidence of contact dermatitis, frostbite, or burns* ○ Vision (i.e. visual acuity and direct fundoscopy) - *looking for signs of optic atrophy* ○ Neurological (i.e., gait, peripheral and cerebellar) - *looking for neurological features of chronic exposure* such as impaired gait, loss of coordination, muscle weakness and peripheral neuropathy)
Hydrogen Phosphide	● Medical history evaluating for past medical conditions or symptoms involving the following systems. ○ Respiratory ○ Haematological – *looking for signs, symptoms and complications of anaemia* ○ Oral maxillofacial - (i.e. history of soft tissue swelling, jaw pain, dental problems and gum discharge) - *looking for evidence of mandibular necrosis* ● Smoking history ● Examination ○ Respiratory – *looking for evidence of bronchitis and respiratory irritation* ○ Neurological examination (i.e., focusing on cranial nerve V, central and peripheral nervous system) - *looking for evidence of motor and speech deficits* ● Specific Diagnostic Tests ○ FBC ○ Spirometry (baseline)
Hydrogen Cyanide	● Medical history evaluating for past medical history and symptoms involving the following system ○ Thyroid ○ Vision ○ Neurological ○ Respiratory ● Examination ○ Neurological examination (i.e. central and peripheral nerve examination) - *looking for non-specific neurological effects such as tremors, weakness and sensory deficit*, ○ Visual examination (i.e., focusing on visual acuity, cover and uncover test and direct fundoscopy) - *looking for features of optic atrophy* and amblyopia ○ Thyroid examination (i.e., goitre) - *looking for evidence of thyroid dysfunction* ● Specific Diagnostic Tests ○ Thyroid Function Tests ○ Urine Thiocyanate Levels
Sulfuryl Fluoride	● Medical History evaluating for past medical history and symptoms involving the following system ○ Neurological symptoms looking for muscle twitching, tingling, numbness or weakness; ○ Gastrointestinal symptoms such as abdominal pain, diarrhoea or constipation; ○ Musculoskeletal symptoms such as pain, muscle weakness, deformity or reduction in range of movement; ○ Dental symptoms such as pain or mottling of teeth ○ Respiratory symptoms such as shortness of breath, cough and reduced exercise tolerance ○ History of past and current alcohol consumption ● Examination ○ Neurological (i.e. gait, central and peripheral nervous system) – *looking for weakness, muscle twitchiness, numbness* ○ Musculoskeletal system - *looking for deformity or stiffness* ○ Abdominal system - *looking for signs of liver disease such as jaundice or hepatomegaly* ○ Dental evaluation - *looking for signs of fluorosis which are mainly chalky appearance, cloudy white lines, brown or yellow discolouration of teeth* ○ Respiratory system - *looking for signs of pneumonitis.* ● Specific Diagnostic Tests ○ Renal Panel ○ Liver Function Tests ○ Urinary Fluoride Level

### Hydrogen phosphide

Hydrogen Phosphide otherwise known as Phosphine or Trihydrogen Phosphide is a colourless gas and has an odour of garlic or decaying fish [18, 19]. Of interest, the level at which humans detect the odour of Hydrogen Phosphide does not provide sufficient warning of dangerous concentrations [18–20]. Exposure to gaseous Hydrogen Phosphide is mainly via inhalation, and is rapidly absorbed and is distributed throughout the body leading to the effects on the respiratory, cardiovascular, and CNS [18].

Hydrogen Phosphide inhibits the body’s ability to produce proteins [18–21]. Furthermore, it has been postulated that Hydrogen Phosphide inhibits cytochrome c oxidase and mitochondrial oxygen uptake. However, this has not been proven in-vivo studies [21]. Most of the absorbed phosphine is excreted in exhaled air and minor amounts are oxidised and excreted in the urine as hypophosphite and phosphate. The biological half-life of phosphine has not been reported and may be difficult to estimate [18, 19]. Of note there are no biological indicators for exposure to phosphine [19].

The chronic effects of long-term or repeated exposure to Hydrogen Phosphide are generally distinct from acute poisoning. Of note, published data on chronic health effects to Hydrogen Phosphide are limited [17]. Several international guidelines have highlighted that health effects due to repeated exposures can result in bronchitis with cough, phlegm, or shortness of breath, anaemia, speech and motor disturbances, toothache, swelling of the jaw, mandibular necrosis, and spontaneous fractures [19, 21–23]. Hydrogen Phosphide is not considered to be mutagenic in vivo. It is not classified as a human carcinogen. No reproductive or teratogenic effects from exposure have been reported [24].

As Hydrogen Phosphide is a respiratory irritant, those with impaired pulmonary function, would be susceptible to the irritant effects of Hydrogen Phosphide [22]. As such, workers who have chronic lung conditions should be deemed unfit for work. Additionally, workers should also be counselled to stop smoking, as it can exacerbate the respiratory effects. For example, a study analysing percentage changes in FEVI and FVC suggested that smoking behaviour and occupational exposure significantly “affected percentage changes in lung function” [25].

Routine medical examination should therefore include (i) history, examination and full blood count evaluating for anaemia; (ii) history of soft tissue swelling, jaw pain, dental problems, and discharge looking for evidence of mandibular necrosis; (iii) neurological examination evaluating for chronic effects of Hydrogen Phosphide such as motor and speech deficits; and (iv) respiratory history and examination (*refer to* Table 1*)*. Workers with abnormal findings should undergo further evaluation. For example, a worker with abnormal findings indicating mandibular pathology should undergo further imaging such as Intraoral (periapical and bitewing) and panoramic radiographs [26].

A baseline spirometry should be done at the pre-licence examination to establish a reference point for future surveillance and assess worker’s respiratory function to ascertain fitness to work [20]. Spirometry should be repeated if worker has abnormal respiratory examination findings, or symptoms from the routine medical examinations. ACOEM proposed that a “confirmed decline of FEV1 of 10–15% as compared to baseline lung function, requires further medical evaluation” [27].

### Hydrogen cyanide

Hydrogen Cyanide (HCN) is a systemic chemical asphyxiant with a bitter almond odour which is often described as having a “musty old sneakers smell” [28–30]. Unfortunately, the odour does not provide adequate warning of hazardous concentrations. Due to its small size and moderate lipid solubility, HCN is rapidly absorbed and distributed into body tissues. Following inhalation, HCN is distributed to the lungs, blood, brain and kidneys. On the other hand, post ingestion, it is found in the stomach or other parts of the gastrointestinal tract mostly [28, 30, 31].

HCN interferes with the normal use of oxygen by nearly every organ of the body and does so via various mechanisms of toxicity. Firstly, cyanide ion blocks oxidative respiration thus causing tissue hypoxia especially affecting the tissues with high metabolic demand such as CNS which are key targets for toxicity [28, 30–32]. Such inhibition of oxidative metabolism gives rise to lactic acidosis. Secondly, cyanide induces the release of neurotransmitters such as N-methyl-D-aspartate (NMDA), resulting in seizures [28, 30–32]. Thirdly, release of biogenic amines also results in the pulmonary and coronary vasoconstriction [28, 30–32].

It is postulated that HCN is irreversibly metabolised via the enzyme rhodanese to the less toxic form of cyanide known as thiocyanate [25, 29]. Rhodanese can be found in mitochondria of tissues and commonly found in liver, kidney, brain, and muscles [28, 32]. Majority of absorbed cyanide is excreted as thiocyanate in the urine while small amounts are excreted unchanged in lungs, saliva, sweat, urine or converted to carbon dioxide in expired air. The plasma half-life of HCN is 2 to 3 h [28, 32].

Chronic exposure may result in non-specific symptoms such as headache, fatigue, and anorexia. Respiratory tract irritation, breathlessness, hoarse voice and chronic rhinitis have also been reported [28, 30–32]. Thiocyanate generated in HCN metabolism is known to disrupt the iodine uptake by thyroid. Chronic exposure to HCN can therefore lead to disruption to thyroid function which includes goitre, and hypothyroidism [30]. A study reported that 35 workers who handled cyanide salts in a cable industry showed evidence of thyroid dysfunction and a positive correlation between serum levels of Thyroid Simulating Hormone (TSH) and thiocyanate [33, 34]. It is therefore important to establish a baseline and routine Thyroid Function Tests (TFT) to identify an increase in trend that may indicate biological exposure of effect from chronic exposure to fumigants [35].

Toxic optic neuropathy has also been observed in some cases of chronic cyanide toxicity, including atrophy, amblyopia, and colour deficits [30, 32]. Toxic optic neuropathy result in progressive loss of visual acuity that usually starts with a blur at the point of fixation, dyschromatopsia, and central scotoma. While slit lamp examination and Goldman visual field evaluation is vital in diagnosing optic neuropathy, screening tests will include assessing visual acuity and direct ophthalmoscopy [36]. HCN has not been classified by IARC as a carcinogen and there has been no epidemiological studies on the reproductive and teratogenic toxicity [37].

Though rare, chronic health effects of HCN include non-specific neurological effects, toxic optic neuropathy and thyroid dysfunction [30, 32]. The components of routine examination would therefore include (i) neurological examination focusing on central and peripheral nerve examination looking for non-specific neurological effects such as tremors, weakness and sensory deficit and (ii) visual examination focusing on visual acuity, cover and uncover test and direct fundoscopy, looking for features of amblyopia and optic atrophy (*refer to* Table 1*)*. Workers with signs indicative of optic neuropathy or amblyopia should be referred to an ophthalmologist for further evaluation. In addition, (iii) thyroid examination looking for evidence of goitre and thyroid function test looking for evidence of thyroid dysfunction should also be undertaken. Workers who have pre-existing thyroid dysfunction should be counselled regarding the health risks of HCN exposure and should be fit to work if workers’ TFT levels are within normal range.

Of note, guidelines from New Jersey, United States of America and Australia recommend the use of urine thiocyanate to detect low-level, chronic exposure to cyanides in the workplace [34, 35] Thiocyanate is a major metabolite of cyanide and has a relatively long half-life of 6–14 days and thus can be used to detect cyanide levels in the body [38]. However, while urine thiocyanate can be used as a biological marker to detect cyanide in the body, its presence of thiocyanate in urine does not necessarily indicate workplace exposure. As non-occupational sources, including tobacco and food such as cauliflower, broccoli, cabbage, and other green vegetables can contribute to urine thiocyanate levels, it is therefore important to take background reference levels into account when interpreting such results [35, 36]. The Health and Safety Laboratory in the United Kingdom stipulate that the background levels of non-smokers should be less than 22umol/L and for smokers should be around < 99umol/L. The reference range for workers exposed to cyanide at workplace, the urine thiocyanate levels should be < 210umol for non-smokers and < 580umol/L for smokers [39].

## Sulfuryl fluoride

Sulfuryl Fluoride (SF) is an odourless and colourless gas which is used to fumigate closed structures [40]. SF is in a gas state at atmospheric pressure. For dispersal into a room or a chamber the liquid is released through an application hose towards a distribution fan, where volatilization occurs rapidly after release [41]. Of note, SF must be contained for a sufficient period of time. Hence, a tent is usually placed around the structure which undergoes fumigation. SF penetrates material quickly and rapidly and dissipates during the ventilation process [40, 42]. Of note, when applicators remove the tent, the gas dissipates to low air levels within 24 h and escapes to lower concentrations. As SF does not cause skin or eye irritation at the concentrations used by applicators, like Methyl Bromide, Chloropicrin is added as a warning agent [40, 41].

The mechanism of toxicity of SF is not well described. It is postulated that SF is toxic primarily through the action of the fluoride ion which inhibits oxygen uptake, disturbs the normal phosphate balance, and inhibited hydrolysis of fatty acids [41]. Of note, animal studies show that the primary route of excretion of SF was via the urine where about 80% of the absorbed dose was excreted by the kidneys [43]. Acute exposure of SF results in nose, eye, throat and respiratory irritation such as pneumonitis and pulmonary oedema. It can also result in neurological symptoms such as numbness, weakness and slowed speech or movements [40–43].

Chronic exposures to SF, a CNS depressant, can cause neurological effects such weakness, muscle twitching, seizures and convulsion. Of note, it can also cause deposits of Fluoride in the bones or teeth leading to fluorosis [44]. Fluorosis may result in pain, deformity, disability and mottling of teeth and bones. Other symptoms involving the gastrointestinal system such as abdominal pain, diarrhoea, constipation; and neurological symptoms like tingling and numbness may also occur [45]. Furthermore, repeated exposures to high concentrations of sulfuryl fluoride may cause liver and kidney damage. Of note, human studies indicate that SG is not a significant irritant nor skin sensitizer [40–44]. Data on genotoxicity, carcinogenicity and reproductive effects are no available [40–42, 44].

Routine examination for SF should include history, examination and specific diagnostic tests. History should screen for (i) neurological symptoms such as muscle twitching, tingling, numbness or weakness; (ii) gastrointestinal symptoms such as abdominal pain, diarrhoea or constipation; (iii) musculoskeletal symptoms such as pain, muscle weakness, deformity or reduction in range of movement; (iv) dental symptoms such as pain or mottling of teeth and (v) respiratory symptoms such as shortness of breath, cough and reduced exercise tolerance. History of past and current alcohol consumption should also be ascertained. It is important that workers are counselled to limit alcohol intake, as it can exacerbate liver damage caused by SF [41].

Examinations on the other hand should be focused on (i) neurological evaluating gait, central and peripheral nervous system; (ii) musculoskeletal system looking for deformity or stiffness; (iii) abdominal system looking for signs of liver disease such as jaundice or hepatomegaly; (iv) dental evaluation looking for signs of fluorosis which are mainly chalky appearance, cloudy white lines, brown or yellow discolouration of teeth and (v) respiratory system looking for signs of pneumonitis [44, 46]. Guidelines from New Jersey, United States of America suggest that liver and kidney function tests are done for fumigators exposed to SF. Furthermore, urine fluoride levels should also be done to ascertain overexposure (*refer to* Table 1*)*. A urine fluoride level of > 4 mg/L is considered overexposure. If overexposure is suspected, patients should also undergo chest X-Ray to look for signs of pneumonitis or pulmonary oedema [44].

## Conclusion

Routine medical examinations allow for doctors to identify and manage possible exposure to fumigants and ascertain workers’ fitness to work. To fulfil such objectives, the components of medical examination should not only be evidence-based but also cost-effective. The fumigants that are commonly used have various health effects. In addition, the risk of developing health effects from exposure to fumigants depend on toxicity of fumigant, exposure intensity, route of exposure and individual susceptibility. As such, it is vital to understand both the nature of the fumigant and circumstances of exposure, to identify the components of medical examination that need to be undertaken.

Aside from medically assessing workers during routine medical examinations, physicians should also counsel workers of the various health hazards posed by the respective fumigants, on the signs and symptoms they should look out for, and to seek help early if warranted. In doing so, physicians can ensure that the negative health implications from exposure to fumigants are kept to the lowest level as practically possible.

## Data Availability

Not Applicable.
